# On boat: A magnificent panorama of River Basin in Tang Dynasty

**DOI:** 10.1016/j.heliyon.2022.e12771

**Published:** 2023-01-07

**Authors:** Wende Chen

**Affiliations:** College of Tourism, Huaqiao University, Quanzhou 362000, China

**Keywords:** Boat, Panorama of River Basin, Historical geography, All tang poetry, Risk factors, Protective factors

## Abstract

Based on the framework of historical geography, this paper studies the panorama of Chinese river basin in Tang Dynasty, and attempts to reveal the connection between boat and panorama of river basin. Research uses logistic regression as method, takes 13,100 five-characters and eight-lines poems in Tang Dynasty as samples to study the influencing factors of boat. Results show that a total of 218 Chinese characters have a statistically significant effect on the factor of boat (P ≤ 0.05), including 141 risk factors and 77 protective factors. This research deepens the historical geography understanding of following nine aspects related to the “boat” in Tang Dynasty: ① waterfront regions, ② natural water systems, ③ aquatic animals & plants, ④ official travel, ⑤ fishery & commerce, ⑥ boat driving, ⑦ wonderful time, ⑧ emotion of boat trip, ⑨ daily life on boat.

## Highlights

• Based on the framework of historical geography, the results of the study provide many new discoveries for historical geomorphology, historical economics, historical animals and plants, and historical culture.
• A large number of samples and calculations enhance the credibility of the study.
• This study not only validates some previous studies, but also deepens historical geography understanding of following nine aspects in Tang Dynasty: ① waterfront regions, ② natural water systems, ③ aquatic animals & plants, ④ official travel, ⑤ fishery & commerce, ⑥ boat driving, ⑦ wonderful time, ⑧ emotion of boat trip, ⑨ daily life on boat.
• The research summarized the huge results and gave a full and literary interpretation.

## 1 Introduction

### 1.1 An important component of Chinese historical geography: River Basin in Tang Dynasty

In order to study the river basin of China in Tang Dynasty (618–907), this paper takes historical geography as theoretical framework of observation. China in Tang Dynasty is an important research field of historical geography, and its core area was the Yellow River and Yangtze River basin. Historical geography provides a correct view of space-time, which is conducive to our understanding of the evolution of places. With the help of this subject, we can observe relatively clear historical clues in the same place. With the help of these clues, we can find some vague space-time. Although there are many records, the Yellow River and Yangtze River basin in the Tang Dynasty are still vague landscapes in historical geography. Considering that this area has always been core area of Chinese civilization, it is worth studying systematically and completely.

As part of the historical geography of the world, Chinese historical geography is an ancient and young subject, which studies the geographical phenomena and the relationship between man and land in the historical period of China. It is ancient because the historical records of China since the Han Dynasty not only focus on the evolution of administrative regions, but also on the description of the relationship between man and land; On the other hand, it is young because modern Chinese historical geography has not been established until 1950s. The former originates from Chinese historical tradition, and the most important representative is Historical Records compiled by Sima Qian (145 BC-?). As we all know, China has a vast “History of the Twenty-four Dynasties”. However, the latter originates from modern geography in the West, and the most important representative is Ratzel Friedrich (1844–1904), who devoted himself to the study of human migration, man-land relationship, and systematically discussed human geography. Semple Ellen Churchill, a student of Ratzel Friedrich, demonstrated the operation of geographical factors in history [1] directly and effectively, which played a key role in the formation of historical geography. Ellsworth Huntington foresaw the importance of climate in history [2]. He regarded climate as a decisive factor for social development, national capacity, ethnic advantage and economic prosperity [3,4]. In modern China, Tan Qixiang (1911–1992), Zou Yilin (1935–1920) and Ge Jianxiong (1945-) are the representatives of historical geography. From the 1950s–1980s, Tan Qixiang had compiled maps of China for dynasties. The Historic Atlas of China [5] has become the foundational work of China's historical geography. Zou Yilin studied the relationship between climate change and agriculture husbandry in history [6], and systematically studied the regional economy in ancient China [7,8]. In addition, he systematically studied the Yellow River and Yangtze River basin in history [9,10], including the canals connecting them [11]. As the most influential historical geographer in contemporary China, Ge Jianxiong has published at least 8 articles on river basin, and his main research fields are focused on the Yellow River and Yangtze River basin [[12], [13], [14], [15]]. He mainly focused on the evolution of the river basin in different historical periods, while the characteristics of the basin in a certain era were not fully studied.

### 1.2 A curiosity about tang: River Basin around boats

“Boat” is chosen as the observation center to study the river basin of the Tang Dynasty for the following reasons: (1) Boats were the most common means of water transportation in the Tang Dynasty; (2) Boats existed in almost all river basins where human activities took place; (3) Ships are not only the carriers of human activities in river basins, but also the gathering places of human activities in river basins; (4) Compared with navigation on rivers, navigation on the sea was extremely rare in Tang Dynasty, and our statistics also show that there is no correlation between “boat” and “sea”, so people in the Tang Dynasty said that “boat” was generally within the river basin. Through the study of ships and their relevance, we have seen a brilliant picture of river basin in that era.

In magnificent Tang Dynasty, boats were the most important means of water transportation, which were the same as horses for land transportation. It is part of history of world transportation as well as history of world tourism. According to the consensus of Chinese, tourism includes six elements of “Food, Hospitality, Travel, Visit, Shopping, Entertaining” [16,17], and travel-mode mainly depends on the transportation of that era. Normally, Tang Dynasty is considered the most exciting era in China, not only because of its economy, but also because of its culture. For most of time, Tang Dynasty was a period with warm climate [18] and beautiful ecology [19]. The research on boats in Tang Dynasty not only helps to understand the travel-mode of that era, but also helps to understand the daily life of Tang Dynasty. Obviously, boat was both a means of transportation and a tool of production. Furthermore, boats are not only necessities, but also witnesses of drifting life in that time.

When people read Tang Dynasty documents, they would find that Chinese Character for “boat” and “horse” appear frequently, which is easy to understand: like horses on land, boat was the most important water vehicle. There are a lot of records about boat trips in Tang poetry, and there are also many records about life on the water. It opens a door of curiosity: How did people travel by boat in Tang Dynasty? What kind of scenery did they see? What did they eat and play on the boat? How were they feeling on the boat? What facilities did the boat rely on? These are enough to arouse human curiosity. Looking for clues about “Boat” in vast literature is certainly one way to understand this curiosity, but we are not satisfied with this: we hope to find some clues that are more closely related to each other. We hope to achieve detective-like work, sorting out a clear chain of evidence from cumbersome clues. Through these, people can fully understand the panorama of the river basin in Tang Dynasty.

## 2 Literature review

### 2.1 Trips in river basin of Tang Dynasty

The Tang Dynasty is a golden age in Chinese history because of its powerful rule and splendid culture [20]. With a population of more than 1 million [21], Capital Chang'an (today's Xi'an) was the most populous city in the world at that time. Its area was seven times the size of Eastern Roman Empire's capital Constantinople, and more than nine times the size of Ming Dynasty capital [22]. Trips in Tang Dynasty can be seen everywhere in the records of various Tang Dynasty documents, especially in Tang poetry, there are many travel poems. Tourism was a way of life, it not only existed in the aristocratic class, but also was daily behavior of ordinary people in Tang Dynasty [23]. According to the Post Office Inscription written by Liu Zongyuan (773–819), a famous writer in Tang Dynasty, with Chang'an as center, there were seven important arterial roads, which radially led to all parts of the country. They connected both land ways and water ways [24]. Trip groups were mainly divided into four categories: first, emperors, nobles and officials, second, intellectuals, third, religious groups (Taoists or monks), and fourth, ordinary people [23]. In addition to above four types of tourists, there are two more common types of tourists: business tourists and inbound tourists [25]. In Tang Dynasty, five regions with rich tourism resources had been formed: two capitals area, Wu Yue area, middle reaches of Yangtze River, Chengdu area and Yonggui (Yongzhou and Guilin) area. At same time, two major tourist resource belts had been formed in Tang Dynasty: Fengshan (Emperor's sacrifice to heaven and earth) Line and Yangtze River Basin Line. Formation of former was mainly influenced by political factors, and formation of latter was mainly influenced by water traffic and tourism endowment [26]. Thanks to the canals constructed by Emperor Yang of Sui Dynasty, important water systems in the country were fully connected, which led to the development of water transportation in that era. People prefer to travel by water rather than land. For example, travelers from Chang'an to Sichuan often take a boat to Jiangdu (today's Yangzhou) first, and then travel up the river to Sichuan [27]. Anyone who understands Chinese geography knows that the distance from Sichuan to Chang'an by land is much shorter than the distance by water, but as Li Bai's famous poem describes: “Road to Sichuan is more difficult than going up to blue sky! [28]" Difficulty of the land road made people choose more predictable waterway.

### 2.2 Shipping routes and boats in Tang Dynasty

**Fig. 1:**
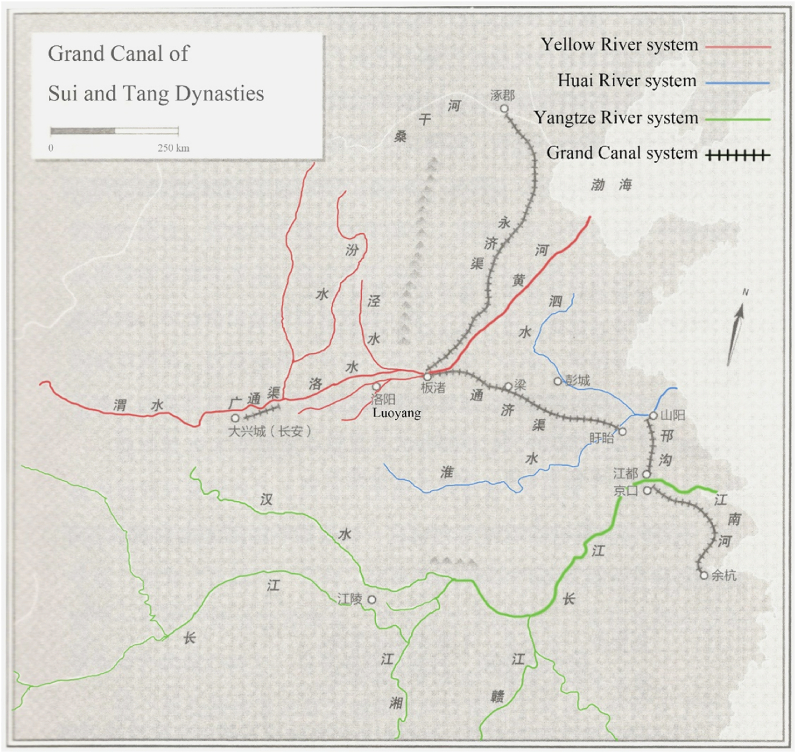
Grand Canal and the three water systems. Editing based on Lewis's original manuscript [31].

Canals digging that took place in Sui Dynasty significantly affected China's water transportation. Apparently, Emperor Yang's motivation for building the canals was to transport materials from southern China to two capitals [29]: a large number of officials and their families lived in Chang'an and Luoyang, and they needed a lot of grain and other products, however, local area could not produce enough products, this forced the emperor to dig canals to connect Yangtze and Yellow Rivers. Through this waterway system, material resources of the south were continuously transported to two capitals. With rapid increase in population of Chang'an in Tang Dynasty, transportation function of the canals became more important in that era [30]. Throughout Tang Dynasty, Yangtze River was one of the most important transportation hubs, and many areas rich in tourism resources were connected by it. Yellow River was the other most important water system, followed by Huai River [27]. Artificially excavated canals connect the above three natural water systems. Fig. 1 shows the relationship between the three river systems.

Six most important tourist routes in Tang Dynasty largely depended on water transportation: ① Fengshan (Emperor's sacrifice to heaven and earth) Line; ② Chang'an-Sichuan Line; ③ From Chang'an to Guizhou (today's Guilin); ④ Canals line; ⑤ Along the Yangtze River; ⑥ Dayuling Line. Five of these six routes are closely related to Yangtze or Yellow River [26,32]. These transports undoubtedly required shipping. Emperors of Tang Dynasty carried out a total of three sacramental activities (Fengshan), and the emperor's team had to walk from Chang'an to Mount Tai, or from Luoyang to Mount Song. It was a national-scale tour that included all important royals, senior officials, and diplomatic envoys from various countries. Travel time was at least two months and a maximum of six months, which had a significant impact on hospitality industry along the way [33]. A large part of Fengshan's journey had to rely on water transportation [32]. Second route was from Chang'an to Sichuan. This route could not all row boats because of Qinling Mountains, but it was possible to row on Wei River and Han River [34]. Routes 3–6 were undoubtedly dominated by water transportation. When Liu Zongyuan was exiled to Yongzhou, he took the third route. Further south along Yongzhou, he went to Guizhou (today's Guilin), the end of third waterway, and wrote many wonderful articles [[35], [36], [37]]. Core of the sixth route is Jiangnan. Original meaning of Jiangnan is south of the middle and lower reaches of Yangtze River [38]. Jiangnan was a newly developed economic and prosperous area in Tang Dynasty [39]. Almost all the great poets in Tang Dynasty wrote poems about visiting beautiful scenery of Jiangnan [40]. The most famous one is “Remembering Jiangnan” written by Bai Juyi. Certainly, literature on fourth and fifth routes is too extensive to list them all.

In 1999, a group of sunken ships were excavated at Liuzi, site of the Grand Canal of Sui and Tang Dynasties, which provided physical information to understand the boats of Tang Dynasty. A total of 8 ancient wrecks were found, including 2 canoes and 6 civil transport ships with wooden planks. The Tang Ship No. 2 was carved and chiseled from a huge camphor wood, with a length of 10.6 m and a diameter of 1.22 m. Main raw material of the other 6 transport ships is also camphor, and Chinese fir wood was used in some places [41]. Tang Ship No. 6 is the largest transport ship, with a length of 27 m and a width of 3.7 m [42]. Further restoration studies show that displacement of Tang Ship No. 6 is 34.2–51.5 tons, and load weight is about 20–38 tons [43]. Because the waterway was shallow, Tang boats were designed with a flat bottom and a round bilge. Such boats had a shallow draft, were less obstructed, and traveled smoothly. When encountering shoal waters, they are easy to pass. Another feature of these Tang boats is flat-headed and square stern, which was a popular style for northern boats at that time [42]. In addition, from technical history, the towing rudder of Tang ship No. 1 enriches history of ship maneuvering technology in the world [44].

### 2.3 Drifting life around river basin in Tang Dynasty

For Tang people, drifting life around river basin could not leave without a boat, which has multidimensional aesthetic imagery [45]: 1. Boats have the beauty of drifting, “lonely boat” and “flat boat” are symbols of impermanent life; 2. Boats have the beauty of parting, as described in Wang Zhihuan's poem: “Don't listen to urging sounds of the oars, otherwise, shallow Peach Blossom Creek will not be able to carry the sorrow of a boat [28]”. 3. Boats have the beauty of freedom, as riding river and sea is a free choice to enter a free world without utilitarianism, furthermore, it is in line with Chuang-Tzu's spirit of A Happy Excursion [46]. 4. Boats have a secluded beauty, just like Confucius said: “If way is not good, I will float on the sea [47]”. 5. Boats have the beauty of ideal realization, because Chinese ancients regarded successful crossing of the river as ideal realization of helping the world. As Li Bai said: “Ship will eventually move forward along the wind and waves, at this time, it should sail into the sea with its sails high [28]”.

Water Channels had gone directly to living quarters of the two capitals. There were five artificial water systems in Chang'an City [48], as there were four artificial water systems in Luoyang City [49]. These water systems met city's life, production, water transportation, flood control, drainage, landscape and other functions [48]. Similar scenes exist in large cities, but Yangzhou is even more special. Everyone in Tang Dynasty knew that in terms of prosperity, “Yangzhou is the first and Yizhou is the second”. At that time, Yangzhou was a fantastic city on the water, “carts and horses are less than boats”, and “neighborhood boats pass by frequently [50]”. In Hangzhou, where economy was booming and population had exceeded 580,000 [51], life on water revolved around the beautiful Qiantang Lake (today's West Lake). At that time, countless pine trees were planted around West Lake. At full moon eve, under translucent silhouette of pine trees, Prefect Bai Juyi saw industrious people working on the water [28].

Fishing in Tang Dynasty was a national industry, as long as there were rivers, there would be fishing [52]. For example, “Hu” is a kind of fishing gear, but it is used as abbreviation of ancient Shanghai, which was already famous for fishing in Tang Dynasty [53]. In Jiangnan area, there were countless such small fishing villages and countless fishermen who specialize in fishing. By the water, there were large and small fish markets [54]. Lu Guimeng wrote a series of poems to describe various fishing gear [28]. For him, that was just an ordinary life. In list of tributes to emperor from all over the country, you can see all kinds of fish [55], and it was not news that royal family likes various styles of fishery products. There were various ways of making fish. The most distinctive food of Tang Dynasty was Kuai, a kind of ancient sashimi, with prepared orange jam Chengji, it became the freshest and most wonderful delicacy. However, using the word “sashimi” is imprecise, as Japanese sashimi undoubtedly originated from Tang and Song China [56].

Development of aquatic animal and plant resources in Tang Dynasty reached a new level. Fish farming industry had developed from a single carp farming to four major fish. The variety and quantity of aquatic vegetables had greatly increased, as quality and planting techniques had also improved. Perch was the star fish described in Tang poems. However, Tang documents mention at least 38 species of freshwater fish, 20 species of marine fish, 30 species of aquatic mollusks, 19 species of crabs, 4 species of shrimp, and 16 other aquatic species animals [57]. Description of plants is almost covered in the literature of Tang Dynasty, because Chinese literature has a tradition of describing plants, such as Book of Songs [58]. Li Jifu recorded 10 huge lakes used to cultivate aquatic plants at that time, with circumferences of 3–50 miles. Main cultivated plants were cattail, reed, thorn grass and edible water chestnut, lotus root and gorgon. Zizania caduciflora, Brasenia Schreberi and Ipomoea aquatica were delicacies commonly found in Tang poetry [57].

When traveling by water, people would often stay at a Waterside Post Station. Post station was a place for officials who passed military information to eat, lodging, and change horses on the way. Stations were often located on the main road, and those located on non-main roads were called Office [59]. In Tang Dynasty, post stations and post offices were set up all over the country, and they were divided into three types: land post, water post and both water and land post [60,61]. Among the famous waterside posts, Xishui Station, Ganshui Station, Fushui Station, Zishui Station, and Changle Office were located in the nearby rivers of Chang'an and Luoyang. Other stations were distributed in Yangtze River Basin and Canal Basin, such as Hengshui Station, Shou'an Office, Haozhou Office, Yangzhou Office, Yiling Office, Penpu Shatou Office, Wuzhou Office [62].

## 3 Methods

Research uses logistic regression to study the correlation between “boat” and other Chinese characters, and uses this as a quantitative method for text analysis. Logistic regression has advantages in exploratory research. It can express the correlation between dependent variables and independent variables in a mathematical way. This research is an exploratory study of quantitative historical geography, which is very suitable for logistic regression method. In the study of nonlinear correlation, logistic regression is the most common and one of the best methods. Feature of Chinese text is that there are correlations between characters, which are nonlinear. To adapt to this feature, this study decided to choose logistic regression as research method. In final model, 309 factors are obtained, some of which are called “risk factors” and others are called “protective factors”. Both of them have a mathematical relationship with the dependent variable “ship”: occurrence of risk factor means that the occurrence probability of dependent variables increases, while the occurrence of protective factor means that the occurrence probability of dependent variables decreases. This study draws on risk and protective factors in medical research methods. In medical research, if a certain factor appears, causing the probability of disease to be higher than normal level, this factor is called a risk factor; on contrary, if a certain factor appears, causing the probability of disease to be lower than normal level, this factor is called a protective factor [[63], [64], [65], [66]]. Of course, this study is not a medical study, but we use the terms risk factors and protective factors in accordance with academic convention.

### 3.1 Correlation between “boat” and other Chinese Characters

Generally, literature of Tang Dynasty that appears “boat” has a high probability of describing life on water. According to this feature, study believes that through the data analysis of literature, it is possible to judge the relevance of “boat” and other Chinese characters, and then to effectively understand the way of life in Tang Dynasty. Therefore, research decided to establish a database including all five-characters, eight-lines poems of All Tang Poetry: study took all 13,100 five-characters and eight-lines poems in All Tang Poetry as samples, and took 6500 Chinese characters of first-level and second-level character lists as variables, established a database containing 84,500,000 data. If a poem just contains the Chinese characters in the variable, the number is displayed as 1, otherwise the data is 0. Drawing on this method, we can find all clues related to “boat”. This design includes a preset: in metrical poems whose main text is fixed at 40 Chinese characters, because each character has been carefully considered by the author, correlation between characters is extremely strong. It is common sense that metrical poetry is a matter of formal decency, and its writing has the strictest and harshest rules. Because of its rigor, author created it by spending a lot of time and energy, which resulted in its stable quality and no overly random writing.

### 3.2 Sample acquisition

A total of 13,100 samples of five-characters and eight-lines poetry are obtained for the study. These samples come from All Tang Poetry on Sinology Navigation website (http://www.guoxue123.com/jijijibu/0201/00qts). Study collects all poems of the same genre as samples. Although their formats are all five characters and eight lines, they can still be divided into two categories: the first type is Ancient Style, which has relatively loose rhyme rules, and the second type is Metrical Poetry, which has stricter rhythm rules. The second type of poetry occupied a dominant position in middle and late Tang Dynasty, which marked the maturity of Tang poetry. The first type of poetry appeared more in middle and early Tang Dynasty, and it was generally considered to be simpler and inherited the ancient literary tradition.

Putting together poems of the same character-count in main body and excluding other types of poems gives the sample a high degree of reliability: it's akin to giving each poet an identically formatted 40-word questionnaire, with only freedom to fill out, so it can be considered that this is an approximately closed questionnaire. Conversely, if two poems of 20 and 200 words are juxtaposed, they are like two vastly different questionnaires filled out by two poets, and there is no point in comparing them.

Research uses its own method to extract samples from Internet. Main logic of the method is the count of Chinese character: the count in main body of poems is the same, and the count of Chinese character in the title of the poems is generally within 1–8, and rarely more than 15. According to this rule, we extract poems with characters within a certain range, and then perform data cleaning. Data cleaning is mainly to remove a few selected other types of poems, followed by removing duplicate poems. In All Tang poetry, repeated poems are generally marked in the title, such as “Yi zuo” (mean “another name”), “You Zuo” (mean “another name”) and so on. After that, in order to ensure pure content for each sample, we also removed the author's name from sample. Through this process, study resulted in 13,100 valid samples.

### 3.3 Data processing

**Fig. 2:**
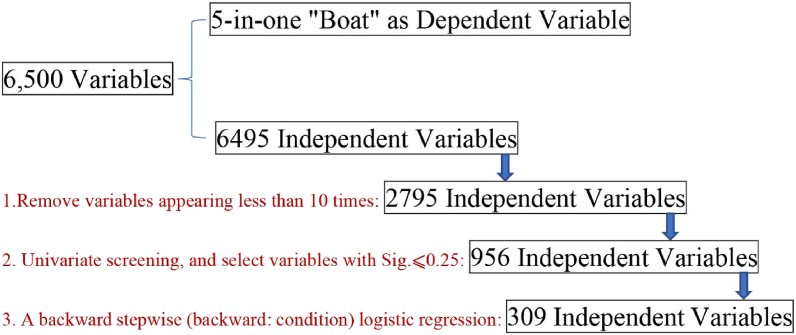
Data processing flow from raw text to final results.

Research takes 6500 Chinese characters as 6500 variables, and finally obtains a model of 309 variables. The data processing flow from raw text to final results is shown in Fig. 2. Measured in these 6500 variables: if the sample contains the corresponding Chinese character, display 1, otherwise, display 0. These 6500 Chinese characters come from First-level and Second-level character lists of Chinese characters, of which First-level character list contains 3500 characters and the Second-level character list contains 3000 characters [67]. In this way, study resulted in a huge Excel table with 13,100 rows, 6500 columns, and a total of 85,150,000 pieces of data. These data only contain 0 and 1. Study removed Chinese characters that appeared less than 10 times, so that the number of variables dropped from 6500 to 2799. Study tended to believe that if certain Chinese characters appeared only 10 times in a sample of 13,100, these characters were of extremely low importance and were excluded from the study.

Study found that in Tang Dynasty, there were five commonly used characters that can be used to express the meaning of “boat”, and study combined these five variables into one variable. These five characters are “Zhou, Chuan, Ting, Ge, Fang”. Among the 13,100 samples, these 5 Chinese characters appear 1044 times, among which, Zhou appears 641 times, Chuan appears 327 times, Ting appears 30 times, Ge appears 29 times, and Fang appears 17 times. This means that 7.97% of Tang poems in this format have descriptions of boats. It can be seen that boats were an indispensable means of transportation in Tang Dynasty.

In this way, research uses logistic regression as method, the five-in-one “boat” as dependent variable, and the other 2795 Chinese characters as independent variable to carry out the next step of research. IBM SPSS Statistics 19 was used for calculations. The calculation formula is as follows. In the formula, $P_{i}$ represents the probability of the event, α Represents the parameter of regression intercept, $\beta_{i}$ represents the regression coefficient of $x_{i}$ (i = 1, 2, …, n), and $x_{i}$ represents the independent variable.

$$P_{i}=F\left[\alpha +\sum_{J=1}^{m}\beta_{i}x_{i}\right]=\frac{1}{1+\exp\left[-\alpha +\sum_{J=1}^{m}\beta_{i}x_{i}\right]}$$

First, we performed univariate screening for each independent variable. This requires 2795 logistic regressions. Through this process, study excluded independent variables with Sig. > 0.25 and selected all independent variables with Sig. ≤ 0.25. Thus, study obtained 956 independent variables. Since study had a total of 13,100 samples, the sample size was 13.7 times that of the independent variable, which was suitable for logistic regression analysis. Selecting independent variables with Sig. ≤ 0.25 in univariate screening is a common practice, which is also justified by statistical practice: it can avoid missing some important variables in final model [68]. Secondly, A backward stepwise (backward: condition) multivariate logistic regression model was used to determine independent variables, with probability of entry and removal as 0.05 and 0.10. Research used 956 Chinese characters as independent variables and “boat” as dependent variable. Significance value less than 0.05 was considered as statistically significant. After a lot of calculations, research finally obtained a model containing 309 independent variables. This means that out of 6500 Chinese characters, less than 309 characters are associated with “boat”. See Appendix 1 for the complete calculation process. Appendix 1 contains a total of 24 process files.

## 4 Results

### 4.1 Model analysis: risk factors and protective factors

In the final model, 309 factors were obtained, and factors with Sig. values less than 0.05 were considered statistically significant. In statistics, Sig. Value is used to determine whether it is statistically significant, and Exp (B) value is used to determine risk factors or protective factors. Among results, factors with an Exp (B) value greater than 1 are called “risk factors”, and factors with an Exp (B) value less than 1 are called “protection factors”. Both of them have a mathematical relationship with the dependent variable “ship”: occurrence of risk factor means that the occurrence probability of dependent variables increases, while the occurrence of protective factor means that the occurrence probability of dependent variables decreases. Results of logistic regression showed that 309 independent variables entered and formed the final model. The complete results are shown in Table 1. Among them, 218 variables have Significance.

**Table 1:** 309 variables in the equation.

	B	S.E,	Wals	Sig.	Exp (B)		B	S.E,	Wals	Sig.	Exp (B)
入	.343	.132	6.726	.010	1.409	砌	−4.550	2.074	4.812	.028	.011
几	−.347	.194	3.189	.074	.707	残	−.482	.219	4.819	.028	.618
刀	−1.509	.961	2.468	.116	.221	轻	.671	.212	9.986	.002	1.956
三	−.311	.167	3.447	.063	.733	界	−1.429	.847	2.848	.091	.240
小	.697	.215	10.455	.001	.733	咽	−1.250	.777	2.587	.108	.287
巾	−1.457	.797	3.343	.068	.233	峡	.807	.288	7.827	.005	2.240
个	1.938	.660	8.612	.003	6.947	香	−.869	.236	13.606	.000	.419
夕	.421	.175	5.808	.016	1.524	秋	.285	.109	6.866	.009	1.330
凡	1.093	.583	3.519	.061	2.984	信	.778	.230	11.476	.001	2.177
门	−.460	.168	7.545	.006	.631	泉	.465	.198	5.487	.019	1.592
之	−.605	.244	6.119	.013	.546	胜	.891	.252	12.499	.000	2.438
子	−.315	.162	3.779	.052	.730	急	.602	.262	5.282	.022	1.826
卫	−2.154	1.152	3.500	.061	.116	姿	−2.583	1.761	2.150	.143	.076
也	−2.402	1.552	2.396	.122	.091	送	.532	.113	22.064	.000	1.702
飞	−.570	.195	8.560	.003	.566	前	.535	.149	12.821	.000	1.708
天	.244	.114	4.563	.033	1.277	洛	.605	.252	5.747	.017	1.831
支	−1.795	.861	4.346	.037	.166	济	1.275	.490	6.777	.009	3.577
不	−.204	.102	4.012	.045	.815	洲	−.422	.256	2.712	.100	.656
车	−1.039	.400	6.764	.009	.354	觉	−.683	.326	4.381	.036	.505
巨	1.519	.547	7.717	.005	4.566	客	.321	.112	8.196	.004	1.379
中	.206	.107	3.728	.054	1.229	扁	8.364	.900	86.436	.000	4290.791
水	.432	.100	18.777	.000	1.540	误	−1.984	1.133	3.068	.080	.137
午	−2.520	1.223	4.245	.039	.080	载	.897	.331	7.315	.007	2.451
今	−.444	.186	5.702	.017	.641	起	.363	.172	4.432	.035	1.437
月	.341	.108	10.000	.002	1.407	盐	1.571	.625	6.319	.012	4.813
户	−.849	.436	3.795	.051	.428	壶	−1.472	.753	3.823	.051	.229
心	−.457	.144	10.068	.002	.633	莲	.915	.279	10.751	.001	2.497
玉	−.605	.267	5.125	.024	.546	莫	.313	.177	3.116	.078	1.367
未	−.553	.161	11.742	.001	.575	荷	.632	.315	4.033	.045	1.881
正	−.886	.269	10.812	.001	.412	晋	−1.720	.823	4.370	.037	.179
去	.274	.118	5.444	.020	1.315	桐	1.024	.436	5.507	.019	2.784
平	.316	.172	3.381	.066	1.372	贾	1.095	.376	8.456	.004	2.988
灭	−1.481	.960	2.379	.123	.227	夏	.702	.231	9.239	.002	2.018
归	.488	.107	20.593	.000	1.629	眠	.961	.239	16.158	.000	2.615
且	.798	.256	9.699	.002	2.222	钱	.683	.349	3.839	.050	1.980
甲	−1.838	1.143	2.589	.108	.159	乘	.509	.255	3.974	.046	1.663
由	−1.174	.570	4.237	.040	.309	秩	2.207	.744	8.798	.003	9.091
史	.795	.326	5.946	.015	2.214	笔	−2.229	.842	7.005	.008	.108
失	−.633	.393	2.596	.107	.531	候	.672	.347	3.752	.053	1.959
丘	1.123	.338	11.007	.001	3.073	躬	2.250	1.061	4.496	.034	9.486
仪	−4.936	3.822	1.668	.197	.007	息	−.761	.451	2.851	.091	.467
白	−.356	.134	7.006	.008	.701	卿	−1.574	.762	4.261	.039	.207
	B	S.E,	Wals	Sig.	Exp (B)		B	S.E,	Wals	Sig.	Exp (B)
瓜	2.763	.601	21.168	.000	15.845	留	.573	.166	11.933	.001	1.774
乐	−.615	.318	3.725	.054	.541	高	−.636	.151	17.767	.000	.529
外	.472	.148	10.097	.001	1.603	郭	1.201	.285	17.741	.000	3.325
市	.925	.328	7.968	.005	2.523	席	1.185	.306	14.967	.000	3.270
头	.382	.197	3.764	.052	1.466	准	3.529	1.278	7.621	.006	34.079
汉	−.493	.235	4.394	.036	.611	旅	.601	.229	6.867	.009	1.823
穴	−2.763	1.176	5.517	.019	.063	料	1.564	.554	7.966	.005	4.776
礼	−1.522	.676	5.072	.024	.218	浦	.830	.206	16.316	.000	2.294
记	−1.881	1.058	3.160	.075	.152	浮	.373	.205	3.330	.068	1.453
出	−.667	.180	13.776	.000	.513	涧	−1.014	.568	3.184	.074	.363
扣	1.890	.681	7.699	.006	6.618	涕	1.467	.618	5.634	.018	4.334
巩	2.038	1.045	3.804	.051	7.678	浪	1.350	.195	47.738	.000	3.856
扬	.921	.318	8.375	.004	2.512	宰	.818	.439	3.467	.063	2.266
臣	−1.971	.605	10.617	.001	.139	被	−3.086	.906	11.597	.001	.046
西	−.345	.160	4.663	.031	.708	陪	.958	.327	8.603	.003	2.607
在	.259	.132	3.850	.050	1.296	萝	−1.799	.652	7.614	.006	.165
成	−.369	.210	3.099	.078	.691	菊	−1.081	.491	4.852	.028	.339
师	−.589	.313	3.547	.060	.555	萍	1.340	.407	10.847	.001	3.818
光	−.355	.212	2.796	.095	.701	梧	−1.507	.907	2.763	.096	.222
当	−.467	.225	4.316	.038	.627	盛	−1.177	.676	3.033	.082	.308
帆	−.432	.200	4.674	.031	.649	鄂	1.496	.590	6.433	.011	4.464
回	.814	.142	33.086	.000	2.257	移	.504	.235	4.612	.032	1.655
仲	1.018	.523	3.779	.052	2.767	偶	.892	.295	9.157	.002	2.441
行	.266	.118	5.058	.025	1.304	停	1.391	.369	14.209	.000	4.019
众	−1.563	.605	6.675	.010	.210	得	−.306	.155	3.913	.048	.736
亦	−.401	.236	2.888	.089	.670	脱	−4.059	2.967	1.871	.171	.017
齐	−1.550	.683	5.157	.023	.212	祭	−5.247	2.179	5.800	.016	.005
关	−.460	.198	5.414	.020	.631	痕	−1.723	.652	6.993	.008	.179
江	.785	.102	59.557	.000	2.192	鸿	−.886	.357	6.168	.013	.412
池	.626	.191	10.774	.001	1.870	淑	1.737	.854	4.137	.042	5.683
讼	2.232	.751	8.831	.003	9.319	淮	.533	.273	3.808	.051	1.704
那	−.852	.428	3.965	.046	.427	渔	1.621	.198	66.809	.000	5.060
欢	−1.166	.432	7.282	.007	.312	梁	.603	.332	3.297	.069	1.828
违	−1.058	.610	3.011	.083	.347	情	−.714	.199	12.841	.000	.490
贡	3.547	.516	47.159	.000	34.699	弹	−1.231	.789	2.435	.119	.292
折	−.746	.431	3.002	.083	.474	绩	2.110	.815	6.706	.010	8.248
孝	1.476	.608	5.895	.015	4.374	维	1.626	.426	14.540	.000	5.084
坟	−2.869	1.143	6.298	.012	.057	越	.976	.210	21.665	.000	2.653
苇	1.578	.492	10.276	.001	4.845	趁	1.547	.700	4.892	.027	4.699
甫	1.539	.473	10.582	.001	4.661	欺	−1.767	1.214	2.118	.146	.171
两	.571	.242	5.573	.018	1.771	蒋	1.704	.702	5.892	.015	5.497
丽	−1.236	.728	2.884	.089	.291	朝	−.457	.187	5.959	.015	.633
轩	−.849	.439	3.732	.053	.428	雁	−.691	.210	10.839	.001	.501
	B	S.E,	Wals	Sig.	Exp (B)		B	S.E,	Wals	Sig.	Exp (B)
步	−.749	.390	3.691	.055	.473	雄	−1.323	.845	2.451	.117	.266
里	.306	.127	5.794	.016	1.357	辈	−2.189	1.008	4.721	.030	.112
园	−.614	.265	5.356	.021	.541	甥	1.753	.919	3.641	.056	5.770
别	.257	.123	4.361	.037	1.292	集	−.883	.499	3.135	.077	.413
我	−.565	.267	4.484	.034	.568	傍	−.980	.462	4.491	.034	.375
低	−.717	.429	2.797	.094	.488	鲁	−2.075	.687	9.128	.003	.126
役	.944	.451	4.379	.036	2.569	曾	−.520	.306	2.890	.089	.594
谷	−.681	.402	2.862	.091	.506	湖	.793	.168	22.305	.000	2.209
邻	−1.190	.432	7.581	.006	.304	渡	.786	.237	10.998	.001	2.195
岛	.667	.248	7.218	.007	1.949	游	.385	.129	8.886	.003	1.470
饭	1.241	.477	6.757	.009	3.459	滋	−1.806	1.259	2.057	.152	.164
系	2.739	.343	63.675	.000	15.471	遍	−.909	.429	4.500	.034	.403
忘	−.598	.376	2.527	.112	.550	登	.369	.196	3.545	.060	1.446
泛	2.024	.206	96.538	.000	7.571	缆	2.252	.542	17.291	.000	9.509
怀	−.457	.209	4.764	.029	.633	瑞	−2.941	1.696	3.009	.083	.053
牢	3.560	.694	26.348	.000	35.156	摇	.519	.288	3.255	.071	1.680
君	−.288	.144	4.012	.045	.750	勤	−1.225	.547	5.017	.025	.294
灵	−.972	.444	4.788	.029	.378	槐	−2.569	1.245	4.259	.039	.077
即	.431	.229	3.522	.061	1.538	碑	−1.253	.847	2.189	.139	.286
层	−1.491	.632	5.571	.018	.225	雷	−1.201	.642	3.494	.062	.301
抽	−3.971	1.960	4.105	.043	.019	蛾	−1.892	1.345	1.978	.160	.151
者	−.717	.312	5.272	.022	.488	锡	1.040	.445	5.466	.019	2.828
英	−1.953	1.192	2.684	.101	.142	猿	−.386	.222	3.040	.081	.679
范	1.279	.592	4.664	.031	3.595	溪	.571	.174	10.767	.001	1.769
苔	−1.267	.405	9.803	.002	.282	墙	−2.742	1.681	2.661	.103	.064
茅	−.968	.500	3.746	.053	.380	裳	−1.912	.808	5.606	.018	.148
枝	−.902	.314	8.226	.004	.406	疑	−.744	.407	3.341	.068	.475
松	−.358	.221	2.634	.105	.699	潇	−1.043	.542	3.705	.054	.353
卖	1.288	.572	5.067	.024	3.624	漫	−1.208	.531	5.174	.023	.299
郁	1.168	.504	5.367	.021	3.215	嘶	−2.096	1.117	3.523	.061	.123
奔	−1.981	1.208	2.688	.101	.138	踪	−1.660	.784	4.482	.034	.190
顷	1.926	.889	4.690	.030	6.862	篇	.899	.388	5.372	.020	2.457
斩	2.969	.827	12.900	.000	19.481	慰	1.366	.467	8.576	.003	3.921
咏	−.800	.323	6.131	.013	.449	燕	−.687	.394	3.041	.081	.503
岸	.846	.170	24.870	.000	2.329	藏	1.396	.298	21.920	.000	4.040
钓	1.315	.211	38.875	.000	3.725	豁	1.848	.860	4.623	.032	6.349
季	1.334	.528	6.390	.011	3.798	攀	−1.083	.600	3.256	.071	.338
岳	.770	.249	9.547	.002	2.161	黯	1.949	.850	5.252	.022	7.022
使	.664	.200	11.033	.001	1.943	囊	−1.750	1.095	2.553	.110	.174
侣	1.103	.370	8.896	.003	3.014	裴	−1.120	.474	5.597	.018	.326
所	−.507	.269	3.558	.059	.602	丞	.728	.309	5.547	.019	2.071
命	−1.086	.515	4.447	.035	.338	旌	−1.377	.539	6.518	.011	.252
鱼	.968	.189	26.309	.000	2.632	渭	−1.526	.615	6.148	.013	.217
	B	S.E,	Wals	Sig.	Exp (B)		B	S.E,	Wals	Sig.	Exp (B)
变	−1.123	.610	3.394	.065	.325	笳	−1.774	1.263	1.973	.160	.170
夜	.272	.120	5.126	.024	1.312	嵩	−1.948	.833	5.467	.019	.143
卷	−.805	.393	4.199	.040	.447	碛	−2.806	1.214	5.342	.021	.060
泊	1.887	.227	69.180	.000	6.601	莎	−3.828	1.997	3.673	.055	.022
沿	1.493	.538	7.693	.006	4.451	晏	−1.724	1.141	2.280	.131	.178
泻	1.911	.652	8.592	.003	6.757	缨	−1.485	.822	3.261	.071	.227
波	.666	.170	15.247	.000	1.945	蹉	−2.222	1.157	3.685	.055	.108
宗	−1.448	.594	5.942	.015	.235	匡	1.652	.498	10.996	.001	5.217
宜	−1.311	.448	8.562	.003	.270	楫	2.741	.378	52.499	.000	15.498
空	−.521	.148	12.468	.000	.594	岘	1.919	.537	12.787	.000	6.811
帘	−1.044	.640	2.662	.103	.352	霏	−5.316	4.378	1.474	.225	.005
试	−.954	.589	2.627	.105	.385	恣	2.060	.720	8.187	.004	7.844
郎	.520	.229	5.131	.024	1.682	貂	2.127	.709	8.998	.003	8.392
诞	2.948	1.212	5.915	.015	19.061	醪	1.734	.830	4.365	.037	5.661
建	.813	.413	3.869	.049	2.255	榭	2.363	.856	7.618	.006	10.625
肃	−2.716	1.933	1.974	.160	.066	蠡	1.524	.683	4.975	.026	4.588
孤	1.359	.132	106.277	.000	3.893	眇	1.982	.751	6.970	.008	7.254
迢	−1.398	.783	3.187	.074	.247	阊	3.062	.832	13.540	.000	21.379
艰	−2.268	1.278	3.148	.076	.104	湍	1.962	.749	6.858	.009	7.111
细	−.980	.404	5.894	.015	.375	杼	2.385	.830	8.247	.004	10.856
指	−1.208	.682	3.135	.077	.299	醺	1.780	.846	4.426	.035	5.930
草	−.441	.158	7.802	.005	.643	婺	2.128	.812	6.876	.009	8.397
胡	−.846	.467	3.279	.070	.429	缥	2.867	1.173	5.970	.015	17.581
荫	2.301	.922	6.228	.013	9.979	肱	2.780	.954	8.489	.004	16.119
柏	−2.728	1.165	5.478	.019	.065	鸱	2.633	.981	7.210	.007	13.920
树	.311	.137	5.160	.023	1.364	涪	1.748	.988	3.129	.077	5.740
潢	3.418	1.239	7.611	.006	30.493	C	−4.154	.179	540.928	.000	.016

**Fig. 3:**
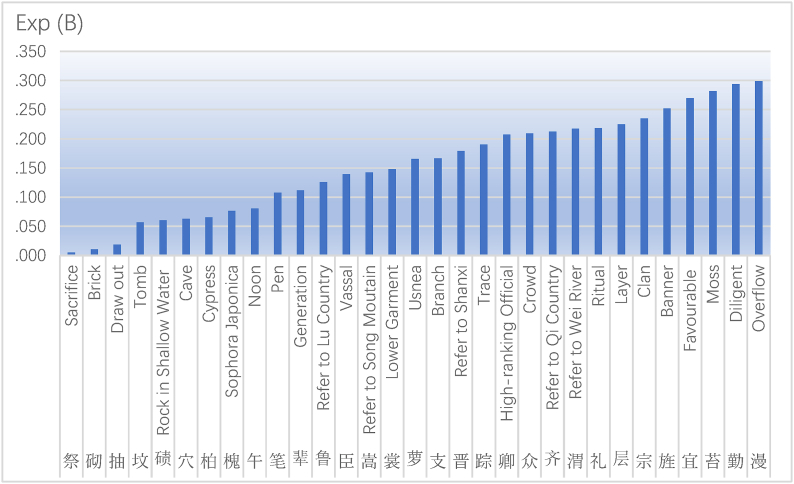
31 protective factors.

The C in the last line refers to Constant values less than or equal to 0.05, and 91 variables have Significance values greater than 0.05. Model passed Hosmer-Lemeshow test, and the Significance value was 0.863, which was greater than 0.05. Proportion of explained variance of model according to Nagelkerke's R2 was 0.544 and Cox & Snell's R2 was 0.229. See Appendix 2 for full results, and see Appendix 3 for last model. The last questionnaire is shown in Appendix 4, and the full questionnaire is shown in Appendix 5. It shows that these 218 variables are statistically significant and have a correlation with the “boat”, while the other 91 variables have no statistical significance with the “boat”. Of the 218 correlation factors, 141 were risk factors and 77 were protective factors. Among the 141 risk factors, there are 20 factors with great contingency. Study eliminated these 20 factors. Table 2 shows the reserved 121 risk factors, which are classified according to the size of Significance value. Fig. 3 shows 31 protective factors with Exp (B) values below 0.3.

**Table 2:**
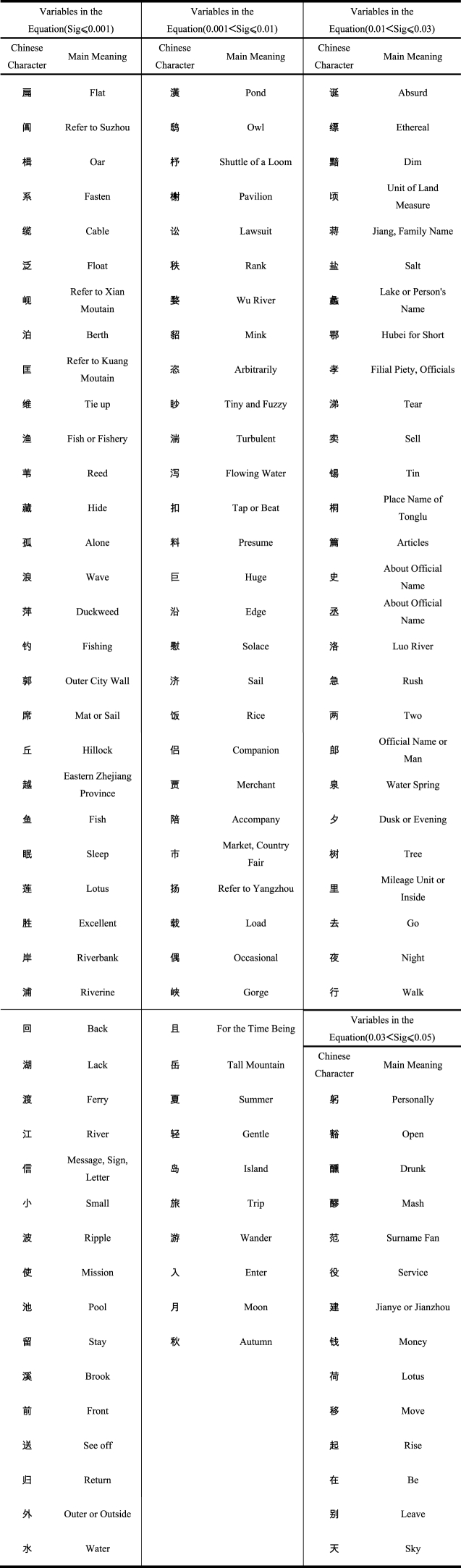
121 risk factors.

### 4.2 Important factors with high exp (B) and low Sig

This study obtained 40 important factors, which were characterized by Odds Ratios higher than 3 and significant values lower than or equal to 0.01, as shown in Table 3. By the way, Exp (B) is equivalent to the Odds Ratios. Due to the above characteristics, there is a high significant correlation between these factors and “boat”, and at the same time, appearance of these factors will greatly increase the probability of “boat”. Obviously, the Odds Ratio of certain factors is extremely high because it often forms a fixed match with the “boat”. For example, “flat” often forms a fixed match with “flat boat”. Another reason is because Chinese character is rarely used, but it is often used to express images related to “boat”, such as “Huang” and “Chi”. Unsurprisingly, we saw a series of images closely related to boats in 40 factors, such as flat boat, oar, cable, berth, sail, and also a series of waterside place names, such as Suzhou, Xian Moutain and Kuang Moutain, while also saw aquatic plants such as reed and duckweed. There are also some terms about the water system, such as Wu River and pond. Of course, as poetry expressing emotion, there are numerous sensuous adjectives, such as alone, arbitrarily, turbulent, and solace, which represent the feelings of people on boat. Although some variables are unexpected, they also make sense, such as lawsuit and rank, which indicate that a large proportion of the boaters' identities were officials. We will interpret them in more detail in Discussion section of this article.

**Table 3:** Important factors with high exp (B) and low Sig.

Sig ≤ 0.001			0.001 < Sig ≤ 0.01		
Chinese Character	Main Meaning	Exp (B)	Chinese Character	Main Meaning	Exp (B)
扁	Flat	4290.791	潢	Pond	30.493
阊	Refer to Suzhou	21.379	鸱	Owl	13.920
楫	Oar	15.498	杼	Shuttle of a Loom	10.856
系	Fasten	15.471	榭	Pavilion	10.625
缆	Cable	9.509	讼	Lawsuit	9.319
泛	Float	7.571	秩	Rank	9.091
岘	Refer to Xian Moutain	6.811	婺	Wu River	8.397
泊	Berth	6.601	貂	Mink	8.392
匡	Refer to Kuang Moutain	5.217	恣	Arbitrarily	7.844
维	Tie up	5.084	眇	Tiny and Fuzzy	7.254
渔	Fish or Fishery	5.060	湍	Turbulent	7.111
苇	Reed	4.845	泻	Flowing Water	6.757
藏	Hide	4.040	扣	Tap or Beat	6.618
孤	Alone	3.893	料	Presume	4.776
浪	Wave	3.856	巨	Huge	4.566
萍	Duckweed	3.818	沿	Edge	4.451
钓	Fishing	3.725	慰	Solace	3.921
郭	Outer City Wall	3.325	济	Sail	3.577
席	Mat or Sail	3.270	饭	Rice	3.459
丘	Hillock	3.073	侣	Companion	3.014

## 5 Discussion

**Fig. 4:**
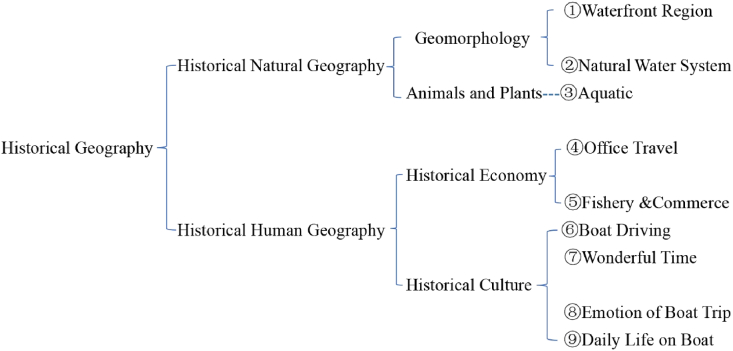
Classification of 9 themes under the framework of historical geography.

In order to explore the panorama of river basin in Tang Dynasty, the study classified the main risk factors according to the theoretical framework of historical geography, and a total of 9 categories were obtained. The induction shows that 9 themes consistently appeared in results, namely ① waterfront regions, ② natural water systems, ③ aquatic animals & plants, ④ official travel, ⑤ fishery & commerce, ⑥ boat driving, ⑦ wonderful time, ⑧ emotion of boat trip, ⑨ daily life on boat. It can be seen from Fig. 4 that ① & ② belong to the category of historical geomorphology, ③ belongs to the category of historical animals and plants, ④ & ⑤ belong to the category of historical economy, and the ⑥ to ⑨ belong to the category of historical culture. Following research will start from these 9 points and discuss the panorama of river basin in Tang Dynasty.

### 5.1 Waterfront regions

**Fig. 5:**
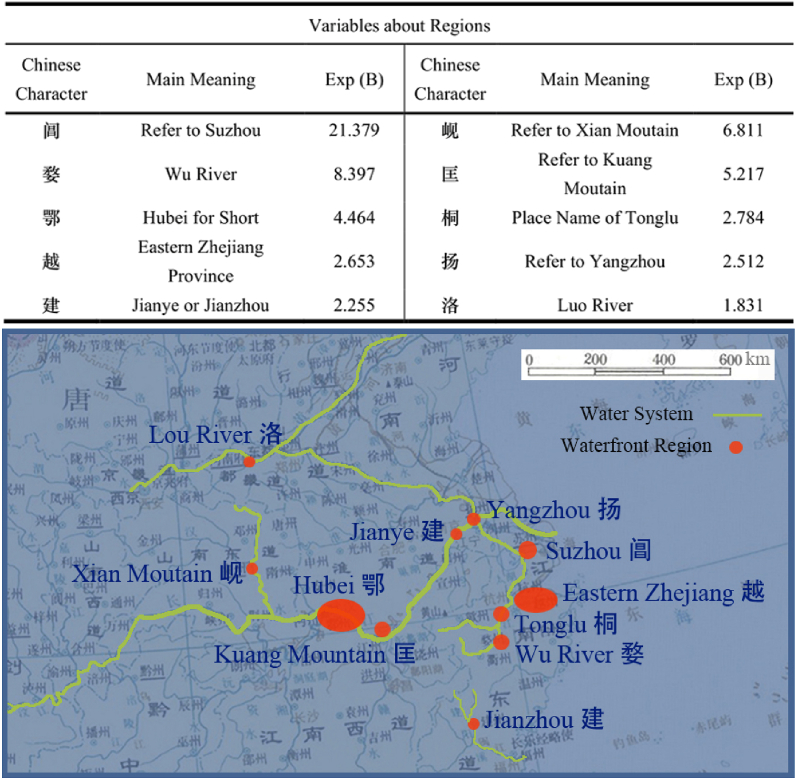
Waterfront regions and the rivers.

Study summarized all place names from results, resulting in a total of 10 factors. Factors with the highest Odds Ratios (ORs) are Chang (Refer to Suzhou), Wu (Wu River), Xian (Xian Mountain), and Kuang (Kuang Mountain), with values of 21.379, 8.397, 6.811, and 5.217, respectively. Obviously, these place names are concentrated in waterfront area, and mainly in middle and lower reaches of Yangtze River. This result reveals that there were at least two water transportation centers in Tang Dynasty, namely Hubei and Jiangnan. Water transportation of Yellow River was mainly concentrated in the middle and upper reaches, with Luoyang as center. Somewhat unexpectedly, Tang people had already explored Zhejiang very deeply, and boat trips had frequently reached Tonglu and Wuyuan. We also examined the factor “Jian” and found that it can refer to “Jianye” or “Jianzhou”. However, from All Tang Poetry, Tang people believed that these two places must be reached by boat commonly, so the study did not remove it. Entering remote Fujian could not avoid traveling by land, but a large part of the journey could rely on water transportation from northern Fujian. Top half of Fig. 5 shows the resulting data of Odds Ratios (ORs), and bottom half shows a waterway map based on results. This picture was drawn by author, and original map was from “The Historical Atlas of China” [5].

### 5.2 Natural water systems

Natural water systems include various rivers, lakes and brooks, as well as flat land and hills on shore, and of course ferry ports, including wetlands near water, islands in water, etc. It is reflected in result model. Table 4 shows various components of the natural water system and their Odds Ratios (ORs) in model. Obviously, these factors can be divided into two categories, one is noun and one is adjective. Most of nouns are related to various natural water systems of the boat, and adjectives are related to various forms of wind and waves on water, such as “Turbulent”, “Rush”, “Flowing”. Interestingly, we have seen many common sights of boats through results, such as “Tall mountain”, “Gorge”, “Riverine”, “Ferry”, “Island” and so on.

**Table 4:** Factors about natural water systems and their ORs.

Chinese Character	Main Meaning	Exp (B)	Chinese Character	Main Meaning	Exp (B)
潢	Pond	30.493	湍	Turbulent	7.111
泻	Flowing Water	6.757	泊	Berth	6.601
沿	Edge	4.451	浪	Wave	3.856
济	Sail	3.577	浦	Riverine	2.294
峡	Gorge	2.240	湖	Lack	2.209
渡	Ferry	2.195	江	River	2.192
岳	Tall Mountain	2.161	岛	Island	1.949
波	Ripple	1.945	池	Pool	1.870
急	Rush	1.826	溪	Brook	1.769
泉	Water Spring	1.592	水	Water	1.540

### 5.3 Aquatic animals & plants

**Fig. 6:**
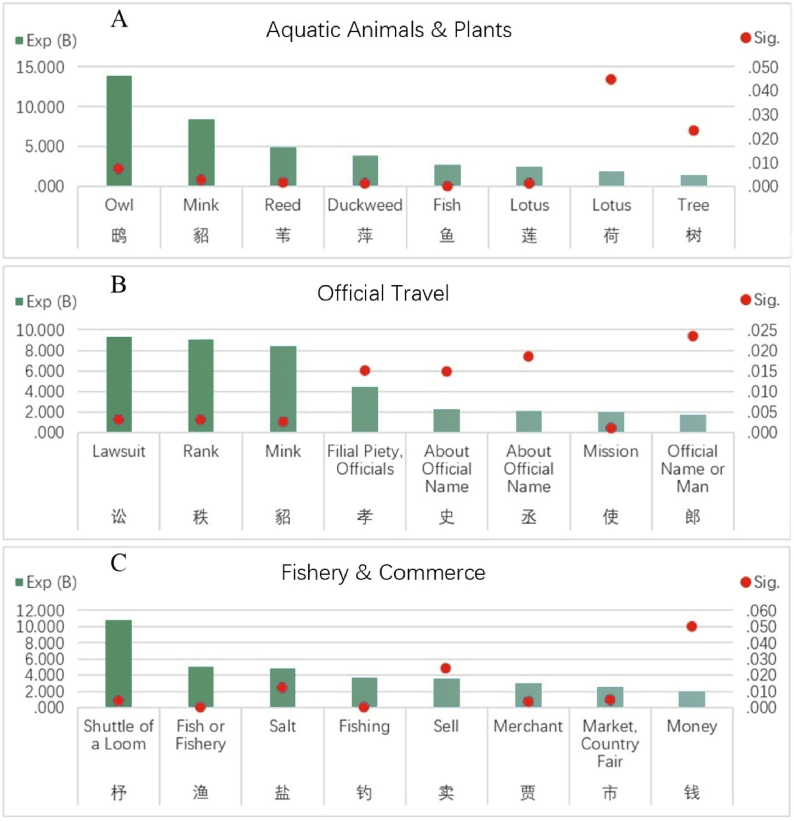
Factors of aquatic animals & plants, office travel, fishery & commerce.

Fig. 6A shows the common aquatic animals and plants on boats in Tang Dynasty. Unsurprisingly, lotus, reeds and duckweeds are frequently found in All Tang Poetry. Common animals are fish and owls, and the more unexpected is mink. Researchers carefully checked the location of “mink” in text, and found that it was often owned by noble people as clothing. It also shows that nobles of Tang Dynasty liked to travel by boat. Ancient Chinese poems have a tradition of praising plants, some plants have moral symbols, and some plants have metaphorical functions. The lotus, for example, is often seen as a symbol of nobility and is often metaphorically referred to as a lover. Xu Yanbo (?-714), a high-ranking official in early Tang Dynasty, described the mood of a boating girl in “Song of Lotus Picking”, also depicted a typical picture of plants in Jiangnan:

The girl who lives by water, sails into the smoky river. When looking for a concentric lover, she harvests the concentric lotus. Lotus root is crisp when broken, and leaves are round when blooming. On this moonlit night, she sings the spring song. When oars returned, flowers are flying ahead [28].

### 5.4 Official travel

Fig. 6B shows the official factor, indicating that Tang officials were keen to travel by boat. Among these factors, many are common official positions, such as Prime Minister, Censor, Secretary General, Inspector General, and some factors refer to official positions in general, such as Xiaolian and Governor, Chinese word Shijun is usually used to refer to a superior officer, which is equivalent to Sir in English. Original meaning of Xiaolian refers to those who are filial to their parents, who are honest and upright. In the Han Dynasty, officials were elected based on this standard. Monk Jiaoran was a descendant of Xie Lingyun, a nobleman of Eastern Jin Dynasty. His poetry was famous for its freshness and naturalness. However, there were also many records of contacts with officials. For example, he wrote it very clearly in following poem:

“Send Secretary Yan to visit East Yue in early spring, and present it to Inspector General Yuan”: Boat is lightly interesting, and east wind blows green fern. If it snows in Mei Fu's seclusion, the Liu family in spring is worth visiting. Wu wine is suitable for mood parting, and Yue people are shocked by the singing. At March meeting in San'in, Governor will get his important assistants [28].

### 5.5 Fishery & commerce

In aforementioned literature review, study mentioned that fishing industry in Tang Dynasty was a national industry, which was very developed. Our research further found that commerce that goes with it was thriving. Fish market by water, salt market by water, merchants by boat, money used for trading, and the fishermen who sell fish, all of which constituted a complete commercial ecological chain. Fig. 6C presents these related elements as a wonderful, long, multipoint perspective picture of life. Surprisingly, as a symbol of the textile industry, shuttle of loom is also a related factor for the boat. We checked the relevant poems and found its rationality: due to development of folk textile industry, it was very common to hear the sound of textile machine beside water. Zhang Ji (766–830), one of the most important poets in mid-Tang Dynasty, wrote this “Hotel at Riverside”, describing the commercial activities on water that he saw during his travels:

As wild Inn facing west wetland, there are orange blossoms ahead the door. Waiting for merchants with lamps, and selling wine to fishermen. Night is quiet, river is white, during the back route, moon is sloping on mountain. As time free, I look for boat to moor, and see smooth sand when the tide ebbs [28].

### 5.6 Boat driving

**Fig. 7:**
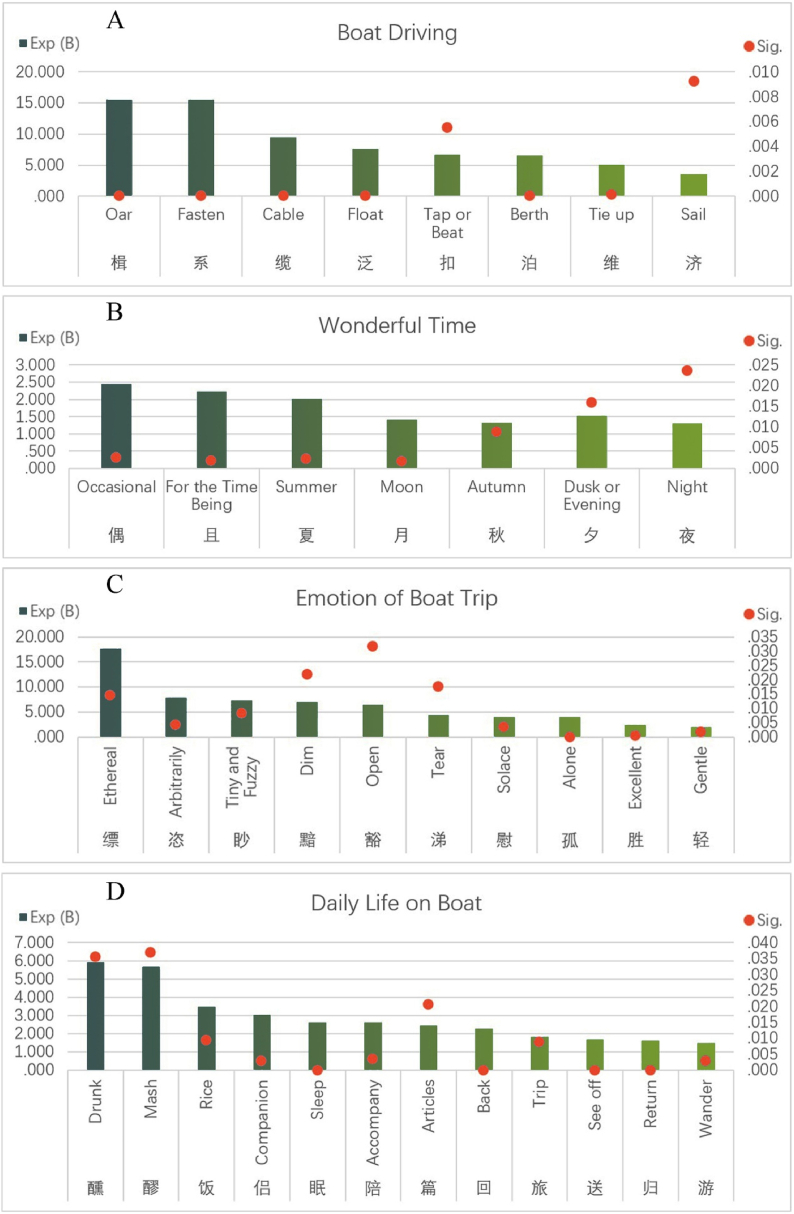
Factors of boat driving, wonderful time, emotion of boat trip, daily life on boat.

A number of factors about boat driving emerged in results, including nouns and verbs. Nouns include oars, cable, berth, and verbs include fasten, float, tap, tie up, and sail. Overall, this series has higher Exp (B) and lower Significance values, indicating that they are significantly associated with boat as results of important factors. Fig. 7A shows the factors about boat driving. Tang Qiu, a poet of late Tang Dynasty, wrote the poem “Berth at Night in Kuizhou”, which describing an unforgettable night in his travels:

Tie up the boat in mirror water, facing White Salt Peak. As might quietly, sand embankment is full of moon, weather is cold, and water temple bell rings. When will the hometown arrive, and when will the old friends meet? If dreaming of returning home, there will be ten thousand layers of green hills [28].

### 5.7 Wonderful time***

As shown in Fig. 7B, 7 time-related factors were present in results. Appearance of two factors, summer and autumn, indicates that summer and autumn should be the main seasons for traveling by boat. It is easier to understand: compared with spring and winter, temperature in summer and autumn is higher, and the river is more stable. Secondly, moon, night and dusk are also common descriptions of time for boating, indicating that dusk and night are the most poetic times for water travel. Of course, these images are usually accompanied by moon or setting sun. It is worth mentioning that moon is one of the most favorite scenes described by poets. Among 13,100 poems in statistics, there are 2696 poems with Chinese character “moon”, accounting for 20.58%. Finally, we see that two Chinese characters “Occasional” and “For the time being” also appear frequently, suggesting a sense of wandering and impermanence in boat trip. Late Tang Dynasty poet Ma Dai (799–869) sailed through Dongting Lake during his exile. Gloomy mood led him to write a touching poem like “Remembering Ancient Times on Chu River”, and also described the beautiful scene of Dongting Lake from dusk to late night: Dew and cold light gather, as the faint sun descends on Chu Mountain. Apes singing from Dongting trees, while men are in a magnolia boat. Bright moon shines on wide water, and green hills are surrounded by turbulent currents. Lord in Cloud haven't come down, making me sad for autumn all night [28].

### 5.8 Emotion of boat trip

Study found that 10 Chinese characters expressing emotions have high Odds Ratios, and the highest among them are “ethereal”, “arbitrarily”, “tiny and fuzzy”, “dim”, “open”, as shown in Fig. 7C. These five are not very common Chinese characters. If the 10 Chinese characters are divided into two categories, expressing inner emotions and situational emotions, the former includes “arbitrarily”, “tear”, “solace”, “alone”, “excellent”, and the latter includes “ethereal”, “tiny and fuzzy “, “dim”, “open”, “gentle”. Li Jiayou's poem “Send Su Xiu to Shangrao” described the pleasant experience of boat trip, which must be due to the fact that he had a similar experience before:

You would be uninhibited, as cloud would be arbitrarily free. Body follows the distant mountains, and lonely boat is left to wander. Less concerned about world affairs, more lodging in fisher's Inn. Boat would be moored at reed flower, while moon on river would shine on you [28].

### 5.9 Daily life on boat

Fortunately, we also see a lot of interesting Tang Dynasty information in results, which shows that boat trip was a daily life that was always about trivial details, while all these details have a warm temperature. As shown in Fig. 7D, the five factors “trip”, “wander”, “see off”, “return”, and “back” indicate that travel is a common event that welcomes and sends. It is worth mentioning that the combination of Chinese “trip” and “wander” means tourism. On the other hand, there were at least 3 items related to food in results, namely “drunk”, “mash of wine” and “rice”, all of which had high Odds Ratios. It seems that for boat trip of Tang Dynasty, while rice was important, wine was even more essential. As a kind of historical evidence, it also shows that in Tang Dynasty China, rice has become the staple food of Chinese people. Appearance of “mash of wine” also indicates that popular wine in Tang Dynasty was turbid rice wine. In addition to these, we speculate that boat trip should be a slightly lonely thing, so “accompany” and “companion” appear in the correlation factors. However, sleeping on boat was also inevitable. Considering that the authors were all literati, they would definitely make full use of their time on boat, so appearance of Chinese character “article” is not surprising. Zhang Ben, a poet in late Tang Dynasty, passed by Suzhou by boat in evening, and wrote “Traveling and berthing in Suzhou”, describing the wine and beautiful scenery that made him indulge:

A boat in Wu River at night, worry is about the sick professor. Whoever perch goes with? The gulls flock themselves. Setting sun is reflecting the water vertically and horizontally, with intermittent clouds in slanted sky. Unlimited thoughts in this foreign land, leaving all mood to the wine, would be just intoxicated [28].

## 6 Conclusion

Within the framework of historical geography, this study has contributed some new insights into the river basin of China in Tang Dynasty, as well as a series of knowledge about the historical scene. This study has strengthened the understanding of the river basin in Tang Dynasty through text mining information about ships. Through this study, the information about the river basin in Tang Dynasty is more abundant, the image is clearer, and the historical truth is further restored. It not only validates some previous studies, but also deepens historical geography understanding of following nine aspects in Tang Dynasty: ① waterfront regions, ② natural water systems, ③ aquatic animals & plants, ④ official travel, ⑤ fishery & commerce, ⑥ boat driving, ⑦ wonderful time, ⑧ emotion of boat trip, ⑨ daily life on boat.

## 7 Limitations and future research

Firstly, as a research method, logistic regression is only applicable to correlation research, not causal research, which means that research is suitable for the initial stage of exploratory research. Secondly, samples of this study did not contain all Tang poetry. This study used all the poems with five-characters and eight lines as samples, a total of 13,100 poems, accounting for about 1/4 of the total number of All Tang Poetry. For other types of Tang poetry, this study has not yet covered. In future research, we should collect samples of more types of Tang poetry for special research or comparative research, such as five-characters & four-lines, seven-characters & four-lines, seven-characters & eight-lines.
